# The Biological Activity Mechanism of Chlorogenic Acid and Its Applications in Food Industry: A Review

**DOI:** 10.3389/fnut.2022.943911

**Published:** 2022-06-29

**Authors:** Liang Wang, Xiaoqi Pan, Lishi Jiang, Yu Chu, Song Gao, Xingyue Jiang, Yuhui Zhang, Yan Chen, Shajie Luo, Cheng Peng

**Affiliations:** ^1^School of Public Health, Chengdu University of Traditional Chinese Medicine, Chengdu, China; ^2^State Key Laboratory of Southwestern Chinese Medicine Resources, Chengdu, China; ^3^College of Pharmacy, Chengdu University of Traditional Chinese Medicine, Chengdu, China; ^4^College of Medical Technology, Chengdu University of Traditional Chinese Medicine, Chengdu, China

**Keywords:** chlorogenic acid, bioavailability, mechanism, source, biosynthesis pathway, food application

## Abstract

Chlorogenic acid (CGA), also known as coffee tannic acid and 3-caffeoylquinic acid, is a water-soluble polyphenolic phenylacrylate compound produced by plants through the shikimic acid pathway during aerobic respiration. CGA is widely found in higher dicotyledonous plants, ferns, and many Chinese medicine plants, which enjoy the reputation of “plant gold.” We have summarized the biological activities of CGA, which are mainly shown as anti-oxidant, liver and kidney protection, anti-bacterial, anti-tumor, regulation of glucose metabolism and lipid metabolism, anti-inflammatory, protection of the nervous system, and action on blood vessels. We further determined the main applications of CGA in the food industry, including food additives, food storage, food composition modification, food packaging materials, functional food materials, and prebiotics. With a view to the theoretical improvement of CGA, biological activity mechanism, and subsequent development and utilization provide reference and scientific basis.

## Introduction

**Figure 1:**
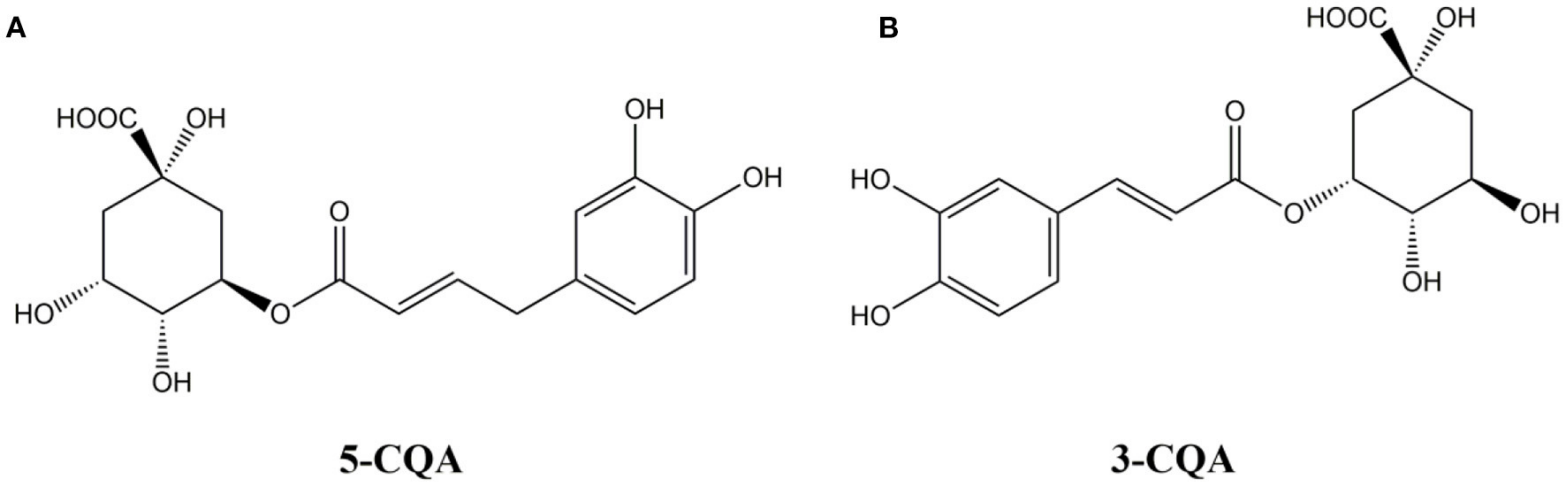
Chemical structural formula of **(A)** 5-CQA and **(B)** 3-CQA.

Chlorogenic acid (hereinafter referred to as CGA), is depside acid produced by caffeic acid (Caffeic acid) and quinic acid (1-hydroxyhexahydrogallic acid). It is a phenylacrylate polyphenol compound produced by the shikimic acid pathway in plants during aerobic respiration. Its hemihydrate is a white needle-like crystal or slightly yellow needle-like crystal, hardly soluble in organic solvents, such as chloroform, ether, benzene, etc., and readily soluble in polar solvents, such as methanol, ethanol, and acetone. According to the different binding sites and numbers of caffeoyl quinic acid, there are ten kinds of chlorogenic acid isomers composed of mono-caffeoyl quinic acid and dicaffeoyl quinic acid, namely:1-coffee Acylquinic acid, 3-caffeoylquinic acid, 4-caffeoylquinic acid, 5-caffeoylquinic acid, 1,3-dicaffeoylquinic acid, 1,4-dicaffeoylquinic acid, 1,5-dicaffeoylquinic acid, 3,4-dicaffeoylquinic acid, 3,5-dicaffeoylquinic acid, 4,5-dicaffeoylquinic acid (1, 2). Among them, 3-caffeoylquinic acid and 5-caffeoylquinic acid are the most widely studied. Their chemical structural formula are showed in Figure 1.

CGA is a natural plant extract with a vast array of sources, present in honeysuckle (3), potato (4), cork (5), *eucommia leaves* (6), *chrysanthemum* (7), strawberry (8), mango (9), blueberries (10), mulberry leaves (11), and green coffee (12). Recently, some researchers have used high voltage discharge to assist in extracting a certain amount of CGA from the three components of tobacco waste (waste, dust, and midrib) (13). *In vitro* and *in vivo* studies have shown that the primary biological activities of the chlorogenic acid are anti-oxidant, liver and kidney protection, anti-bacterial, anti-tumor, regulation of sugar metabolism and lipid metabolism, anti-inflammatory, and protection of the nervous system. Table 1 summarizes its biological activity mechanism.

**Table 1:** The biological activity mechanisms of CGA (↑increase/enhance,↓decrease/inhibit).

**Biological activity**	**Mechanism of action**	**References**
Anti-oxidant	↓Active oxygen (ROS)	(14)
	↓Keap1 ↑Transcription factor erythrocyte derived 2 related factor 2 (Nrf2) signaling pathway	(15)
	↑Superoxide dismutase (SOD) ↑Catalase (CAT) ↑Glutathione Peroxidase (GSH-Px) ↑Glutathione (GSH) ↓Malondialdehyde (MDA)	(16)
	↑Heme oxygenase (HO-1)/Quinone oxidoreductase-1 (NQO-1)	(17)
	↑Endothelial Nitric Oxide Synthase (eNOS)	(18)
	↑Taurine upregulates gene 1 (LncRNA-TUG1)	(19)
	↑*p*21^Waf1/Cip1^ (*p21*)	(20)
	↑Sirtuin1 (SIRT1) ↑AMPK phosphorylation ↑Co-activated receptor factor (PGC-1)	(21)
Protect liver and kidney	↑*ERK*1/2 phosphorylation	(22)
	↑Glucose regulatory protein 78 (GRP78) ↑C/EBP homologous protein (CHOP) ↑Glucose Regulatory Protein 94 (GRP94)	(23)
	↓Caspase-9/Caspase-3	(24)
	↑Hepatocyte Growth Factor (HGF)	(25)
	↓Tumor Necrosis Factor-α (*TNF-α*)/Interferonγ (*IFN-γ*) ↓TLR4 ↓*IRAK-1* ↓*MAPK* signal pathway	(26)
	↓Interleukin 1β (*IL-1β*)/Interleukin 6 (*IL-6*)	(27)
	↓Inducible Nitric Oxide Synthase (iNOS)	(28)
Anti-bacterial	↓Gene LPxB/LPxC ↓Lipopolysaccharide (LPS)	(29)
	↓Succinate dehydrogenase (SDH)/malate dehydrogenase (MDH)	(30)
	↓Extended-spectrumβ-lactamases (ESBLs)	(31)
	↓Quorum Sensing (QS)	(32)
	↓Tricarboxylic acid cycle (TCA) ↓Glycolysis	(33)
Anti-tumor	↓Anti-apoptotic gene *Bcl-2*/*Bcl-XL* ↑Pro-apoptotic gene *Bax*/*Bcl-XS*/Bad	(34, 35)
	↑p38 mitogen-activated protein kinase (p38 *MAPK*) ↑c-Jun N-terminal Kinase (*JNK*) ↓Stem cell marker genes *Nanog, POU5F1, Sox2, CD44, Oct4*	(36)
Anti-tumor	↑*P53*	(37)
	↑Small Ubiquitin-like Modifier 1 (SUMO1) protein ↑*p21*	(38)
	↓*MAPK*/*ERK* activation	(39)
Regulation of carbohydrate and lipid metabolism	↑CuZnSOD (*SOD1*)/MnSOD (*SOD2*) ↓Glycosylation end products (AGEs) ↓*ERK* signaling pathway	(40–42)
	↓Fasting blood glucose (FPG) ↑Insulin sensitivity	(43)
	↑Phosphatidylinositol-3-hydroxykinase (PI3K) phosphorylation ↑Insulin receptor substrate (IRS-1)	(44)
	↓α-Glucosidase (α-GLU) ↓α-amylase	(45)
	↑Glucose Transporter 2 (*GLUT2*) ↑Phosphofructokinase (PFK)	(46, 47)
	↑High-density lipoprotein (HDL) ↑Carnitine palmitoyl transferase (CPT) ↓β-hydroxy-β-methylglutamyl-CoA (HMG-CoA) reductase ↓Fatty acid synthase (FAS)	(48)
	↑Hormone-Sensitive Lipase (HSL)	(49)
	↑Fat triglyceride lipase (ATGL) ↓Transcription Regulator (SREBP-1c/LXRα) ↑ACO (key rate-limiting enzyme for liver fatty acid beta oxidation)	
	↓Lipogenic enzymes	(50)
Anti-inflammatory	↑NF-κB signaling pathway	(51)
	↑Nrf2/HO-1 signaling pathway	(52)
	↓Cyclooxygenase-2 (COX-2)	(53)
	↓Toll-like receptor 4 (TLR4) and its downstream signals (including IRAK1 and TRAF6) ↑Tollip/RP105/SOCS1 mRNA expression	(54)
	↓p38 *MAPK* signaling pathway ↓*TNF-α*/*IL-1β*/*IL-6*	(55)
Protect the nervous	↑Akt/mammalian target of rapamycin (mTOR)pathway	(56)
system	↑mRNA of internal cortisol marker CD31 ↓Expression of vasoconstrictor (ET-1) mRNA	(57, 58)
	↑Cathepsin D protein (an aspartic protease important for lysosomal proteolysis) ↓mTOR signaling pathway	(59)

Recently, as the adverse effects of synthetic substances on the human body have been unveiled, people have paid more attention to the active ingredients in natural plants. Phenolic acids have received massive attention recently due to their good nutritional functions and biological activities such as anti-oxidants (60, 61). Not only has it played an extremely vital role in the field of medicine, its contribution in the field of food is also recognized. As science and technology advances and research deepens, the biological activity mechanism and application of CGA have gradually been noticed. This study, focuses on discussing the biological activity and mechanism of CGA and its application in the food field, hoping to provide a reference for the theoretical improvement of the biological activity mechanism of CGA and subsequent development and utilization.

## The Source of Chlorogenic Acid

**Figure 2:**
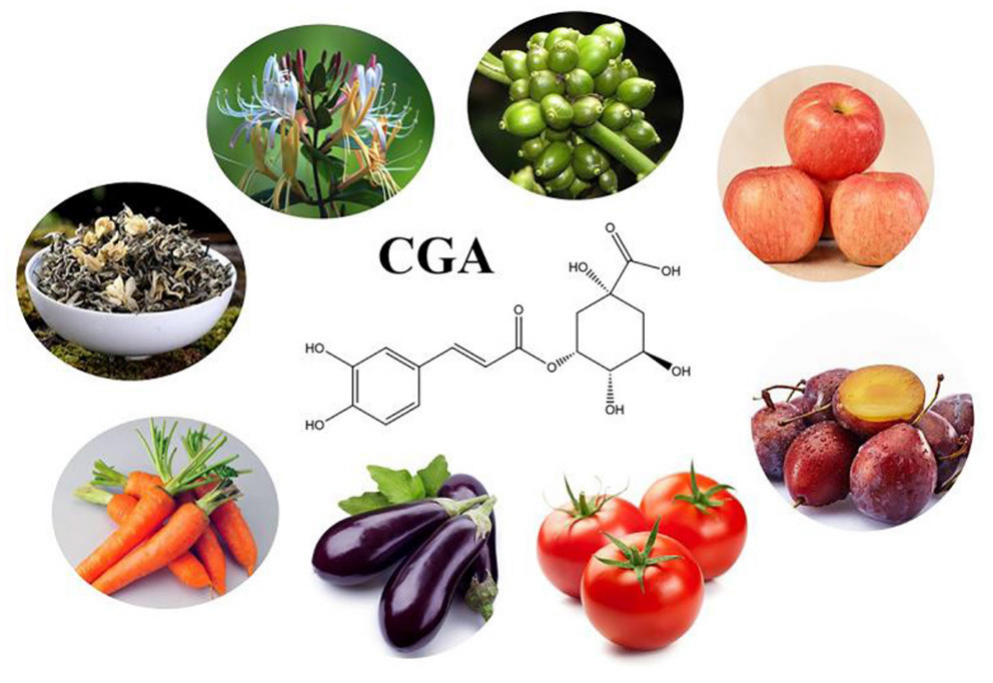
The main source of CGA.

CGA is a phenolic compound produced by plants, and the content in green coffee is very significant, of which 5-CQA is the most abundant, accounting for 50% of the total CGA in green coffee beans (based on dry weight) (12). Unroasted coffee beans are as high as 543.23 (mg/L), but with the increase of roasting degree, the content gradually decreases (62). In addition to coffee, fruits and vegetables as well as Chinese herbal medicines are also a wide range of sources of CGA. The level of CGA in apple is 0.41–1.16 mg/g; prunes contain high levels of CGA (1.3–3.9 g/100 g) and neochlorogenic acid (39.8–92.0 g/100 g) (63). The level of CGA in tomato is 21.30–240.16 ug/g (DW) (64). CGA is the main phenolic compound of eggplant (1.4–28.0 mg/g) and accounts for 80–95% of the total hydroxycinnamic acid present in the pulp (65). The level of CGA in carrot is 0.3–18.8 mg/g. The results of an experiment analyzing the content of phenols, carotenoids and sugars in Vietnamese tea and herbal tea showed: 1 g jasmine tea contains 56.89–307.30 ug CGA, and 1 g green tea contains CGA as high as 219.49–250.41 ug (66). CGA is also considered to be the most characteristic and indicative constituent of honeysuckle (3). The common sources of CGA are showed in Figure 2.

## Chlorogenic Acid Biosynthesis Pathway

CGA is relatively common in plants and fungi. In view of the good biological activity of CGA, it is of great significance to improve human life, which is mastering the biosynthesis pathway of CGA and improve the content of chlorogenic acid in medicinal plants and fungi by means of biotechnology. Comprehensive literature analysis, the biosynthesis of CGA mainly depends on the following three key enzymes: Phenylalanine ammonia-lyase (PAL)/Shikimic acid/Quinic acid hydroxyl cinnamyl transferase (HCT), and Quinic acid cinnamate hydroxyltransferase (HQT). Increasing the concentration of these enzymes can greatly increase the concentration of CGA (67, 68).

**Figure 3:**
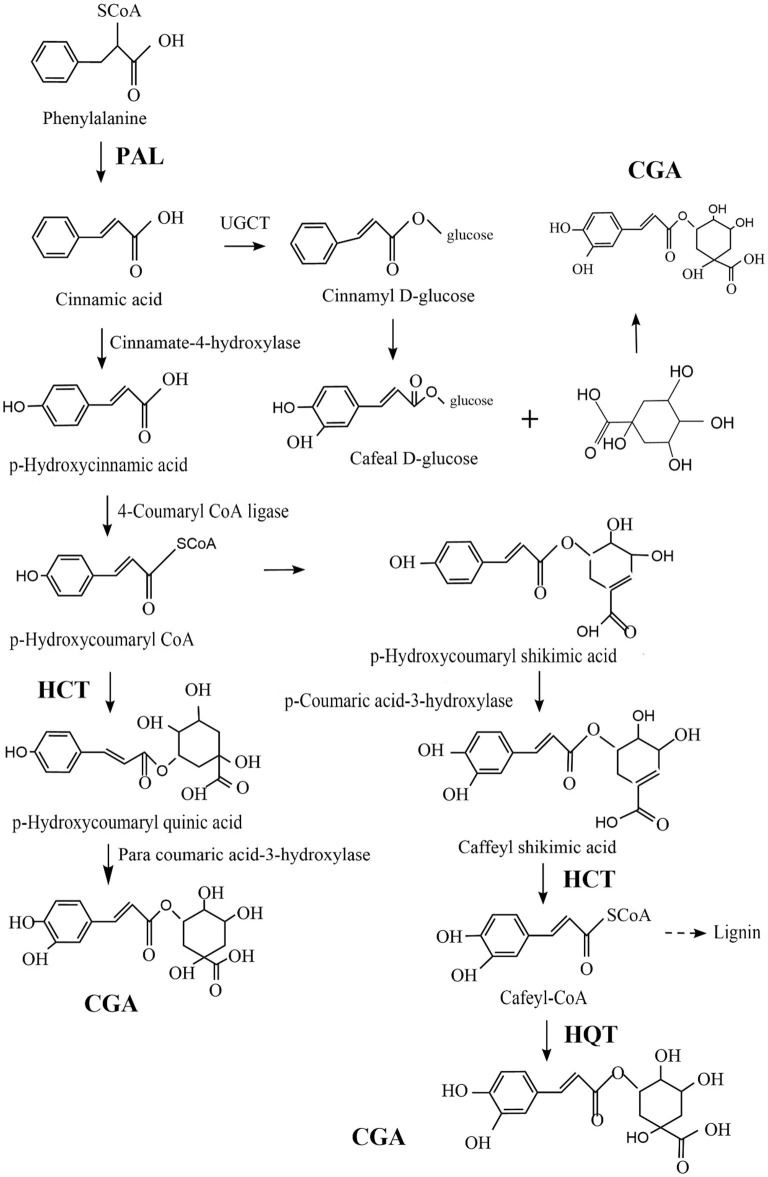
Three possible pathways of CGA synthesis.

PAL dissociates ammonia from L-phenylalanine to produce trans-cinnamic acid, which is a key rate-limiting enzyme (69–71), responsible for the transformation of phenylalanine to trans-cinnamic acid, and is a channel from primary metabolism to phenylpropane-like secondary metabolism (72). Acylation is an important step in the generation of secondary metabolites, Under the catalytic action of HCT, p-Hydroxycoumaryl-CoA provides acyl to quinic acid/shikimic acid, to form p-Coumaryl-quinic acid/shikimic acid, which is the substrate of hydroxylated cinnamyl transferase (C3H) (73). HCT can also remove shikimic acid from the C3H catalyzed product and convert it into Cafeyl-CoA. HQT and HCT both belong to the acyltransferase family. Different from HCT, HQT has acyl-receptor specificity and is the enzyme catalyzing the last step of CGA biosynthesis, catalyzing the transesterification of Cafeyl-CoA and quinic acid to generate CGA (74). Figure 3 shows three possible synthetic pathways for CGA.

## Bioavailability of CGA

There are individual differences in the absorption and metabolism of CGA, and the dose, duration, organs, cell typesand inter-individual differences may all be factors that affect the degradation of CGA. The results of the current study indicate that CGA is absorbed through at least two routes. One route may be immediate absorption in the stomach or upper gastrointestinal tract, and the other route may be slowly absorbed throughout the small intestine. The absorbed CGA is either completely absorbed and utilized, or hydrolyzed to further combine with sulfate, glucuronic acid, or methyl group, or it may be hydrogenated, α/β oxidized, or metabolized by the intestinal microflora (75). An experiment on CGA bioavailability found that in urine, the recovery rate of CGA was only 0.8%, and CGA metabolism through microbial pathways accounted for 57.4% of the total initial intake of CGA, such a high abundance of microbial metabolites. It indicates that the bioavailability of CGA is closely related to the metabolic capacity of the organism's gut flora (76). This finding provides research directions for malabsorbed polyphenols.

## The Biological Activity and Mechanism of Chlorogenic Acid

### Anti-oxidation

**Figure 4:**
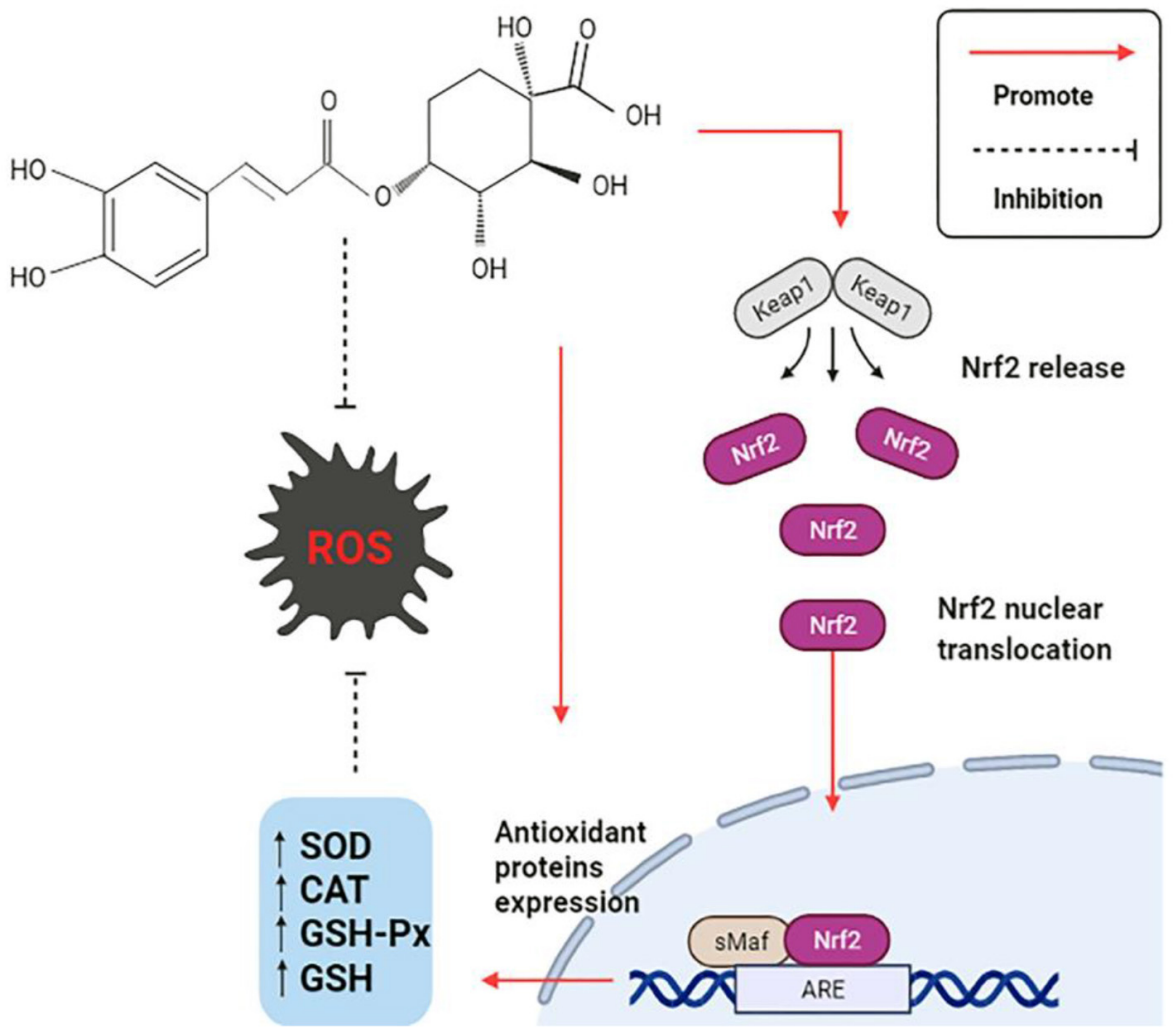
Anti-oxidation mechanism of CGA.

The production of free radicals, often accompanies the daily physiological activities of the human body. Once the free radical's content significantly exceeds the range of anti-oxidant tolerance, a series of abnormal changes will be stimulated in the body. Oxidative stress is caused by the disruption of the balance of the anti-oxidant system in the body, which is the imbalance between reactive oxygen species (ROS) and the anti-oxidant defense system (77). CGA has a comprehensive anti-oxidant mechanism, is summarized as follows: (1) The polyhydroxyl structure directly scavenge free radicals; (2) Activate anti-oxidant signaling pathway, regulate the expression level of related genes, and enhance anti-oxidant capacity; (3) Directly regulates the activity of endogenous oxidase system and associated proteins. The anti-oxidant mechanism is shown in Figure 4.

The natural anti-oxidant performance of CGA depends on its unique molecular structure, which contains five active hydroxyl groups and one carboxyl group. The phenolic hydroxyl structure readily reacts with free radicals and form hydrogen free radicals with anti-oxidant effect, eliminating hydroxyl radicals and superoxide anions as well as exhibiting a strong anti-oxidant effect (14). It was found that with the help of polyhydroxyl structure, CGA could inhibit hydroxyl groups from attacking sulfhydryl groups and be transformed into intramolecular or intermolecular disulfide bonds. CGA can also form protein-polyphenol complex by hydrogen bonding with protein, forming a thicker interface layer, inhibiting the diffusion of free radicals, and enhancing the anti-oxidant capacity of protein. Simultaneously, a large number of free radicals are generated when the oxidation system attacks the side chain residues of amino acids, oxidizing tyrosine monomer into dityrosine, while CGA reduces the free radicals and the contact with the residues, thus preventing the formation of tyrosine dimer (78). Chronic exposure to cadmium (Cd) can cause significant damage to the liver and gastrointestinal tract. Some researchers selected pure CGA reagent and sunflower seed extract (SSE) for treat cadmium-exposed rats, and high concentrations of Cd were detected in the feces of the rats. Preliminary judgment: Chlorogenic acid is a multi-site ligand with several metal complexing sites, especially carboxyl and catechol groups, CGA and SSE complexes may form chelates with Cd, reducing its intestinal absorption (79).

CGA directly or indirectly promotes the expression and activation of anti-oxidant signaling pathways, thus exerting a strong anti-oxidant effect. In the study of CGA on cerebral ischemia/reperfusion (CI/R) rats, it was discovered that CGA reduces the expression of Keap1, an important regulator of cellular oxidative stress response, and activates the most sensitive transcription factor erythrocyte derived 2-related factor 2 (Nrf2) signaling pathway (15). Keap1Nrf2/ARE signaling pathway decreases malondialdehyde (MDA) levels by upregulating the mRNA expression of a series of endogenous anti-oxidant enzymes (16), such as superoxide dismutase (SOD), catalase (CAT), glutathione peroxidase (GSH-Px), glutathione (GSH), etc. Meanwhile, it promoted the expression of downstream protein heme oxygenase (HO-1) and NAD (P)H: quinone oxidoreductase (NQO-1) in Nrf2 pathway, and significantly limited CI/R induced oxidative stress (17). The damage of the intestinal epithelial barrier caused by the weaning stress of piglets can easily lead to various gastrointestinal diseases in piglets, which causes considerable economic losses to animal husbandry. The piglets were fed with CGA-supplemented diet for 14 days, and the differences were compared with or without CGA. The results showed that the activities of GSH-Px and CAT were increased in the duodenum, but the activity of SOD and the content of MDA were not affected; GSH-Px and CAT were also increased, and MAD content decreased in the jejunum, but there was no significant difference in SOD; CGA increased CAT and SOD activities and decreased MDA content in ileum, but there was no significant effect on GSH-Px. Nrf2 played a crucial role in regulating the levels of endogenous antioxidant enzymes that fighted oxidative stress. CGA had also been shown to increase Nrf2 mRNA levels in the small intestine of weaned piglets, which was consistent with the above mentioned results of increased oxidase activity. HO-1 is an important antioxidant enzyme regulated by Nrf2, and previous studies have also reported that CGA can alleviate oxidative stress by increasing the level of HO-1. In conclusion, CGA improves the antioxidant capacity of weaned piglets. The reason may be related to the enhanced activity of antioxidant enzymes mediated by Nrf2/HO-1 signaling pathway (54).

A study to delay vascular aging *in vivo* and *in vitro* also found that CGA inhibits endothelial cell aging through the Nrf2/HO-1 pathway. Nrf2 translocateed into the nucleus more rapidly in CGA-treated cells, and increasing HO-1 mRNA and protein expression, which in turn rapidly reduces oxidative stress, leading to attenuated vascular aging. Using a specific inhibitor of HO-1 (zinc protoporphyrin IX, ZnPP) in experimental mice induced by angiotensin II, it was found that ZnPP could reverse the beneficial effects of CGA. These facts all demonstrated that the beneficial effects of CGA on endothelial cell senescence depended on the regulation of the Nrf2/HO-1 pathway (18). In addition, Nrf2 protein was found to act as a binding partner of taurine up-regulated gene 1 (IncRNA-TUG1) and was positively regulated by IncRNA-TUG1. CGA could promote the expression of IncRNA-TUG1 both *in vivo* and *in vitro*, which in turn indirectly promoted the expression of Nrf2 protein and protected cells from oxidative stress damage (19). *P21*Waf1/Cip1 (*P21*) also plays a vital role in promoting the activation of the Nrf2 pathway. Depletion of *P21* is often accompanied by the blocking of the Nrf2 pathway, while CGA can promote the activation of Nrf2/HO-1 pathway by reversing the downregulation of *P21*, thus making it serve a continuous anti-oxidant role in oxidative stress (20).

CGA can also directly regulate the expression of antioxidant enzyme mRNA and change the activity level of related proteins. Aluminum (Al) exists widely in the environment, and a large amount of accumulation in the human body will cause serious toxicity to the body. The researchers found the level of MDA was increased and the activities of SOD, CAT, and GSH were also significantly decreased in AlCl_3_-induced RAW264.7 cells. However, after CGA (150 μg/mL) treatment, compared with AlCl_3_ alone, the MDA level in the cells was not only reduced by 32.4%, but the SOD, CAT and GSH levels were also increased by 68.9, 45.3, and 39.7%, respectively, which suggested that CGA attenuated the immunotoxicity of AlCl_3_ to cells by increasing their antioxidant capacity (77). Oxidized low-density lipoprotein (oxLDL) has been reported to induce cellular senescence in blood vessels by promoting oxidative stress, resulting in deterioration of the cardiovascular system. Sirtuin1 (SIRT1) is a mammalian homolog of yeast silencing information regulator 2 (Sir2) and is deemed an essential promoter of anti-oxidant genes (80). CGA can reverse the phosphorylation of OXLDL-inhibited protein kinase (AMPK) and the expression of coactivator (PGC-1) by activating SIRT1, as well as maintain the stability of the mitochondrial membrane potential of human endothelial cells, decrease the production of ROS, and ease oxLDL-promoted atherosclerosis to some extent (21). Additionally, CGA also has an excellent protective effect on the oxidative stress induced by 4-tert-octylphenol (OP), which reduces the anti-oxidant capacity of cells, thus causing anemia and clinical symptoms of mineral disorders. Experiments have shown that CGA can increase the expression of SOD/CAT/GSH mRNA to resist the toxicity of OP (81).

### Protect Liver and Kidney

**Figure 5:**
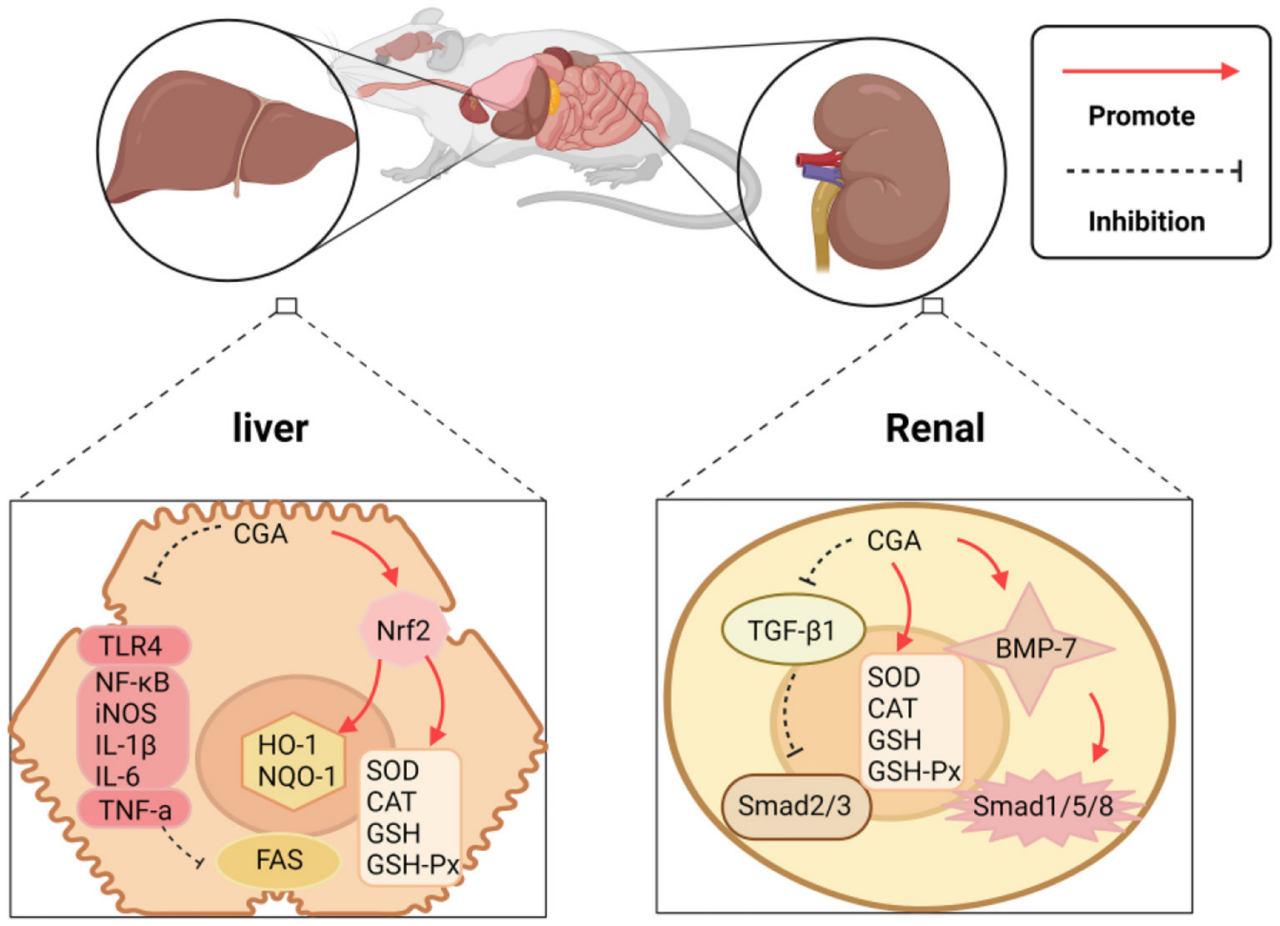
CGA protects the liver and kidney mechanism.

As the absorption and conversion stations of the human body, the liver and kidney play one of the most critical roles of the human body. The liver and kidney damage will bring a double burden on the human body, both mentally and physically. CGA has been proven to have a good protective effect on the liver and kidney, and its protective mechanism can be summarized as the following: (1) It acts on the expression of enzymes and proteins related to the oxidative system and inhibits liver and kidney damage caused by oxidative stress; (2) The regulation is related to apoptosis. The expression level of the gene promotes apoptosis of necrotic cells; (3) Directly or indirectly inhibits the expression of pro-inflammatory factors and related signal pathways. The mechanism of protecting the liver and kidney is shown in Figure 5.

CGA's protection of the liver and kidney can be embodied in regulating the expression of enzymes and proteins related to the oxidation system, thereby restricting liver and kidney damage caused by oxidative stress. Reports indie that CGA protects against drug-induced acute liver failure caused by acetaminophen (APAP) overdose. The mechanism of action is to induce the continuous phosphorylation of extracellular regulatory protein kinase 1/2(*ERK1*/2), and *ERK1*/2 regulates CGA induction as the Nrf2 signaling pathway plays a crucial role in transcriptional activation. CGA activates the Nrf2 anti-oxidant signaling pathway by blocking the binding of Nrf2 to its inhibitor Keap1, and regulates the expression of the downstream genes HO-1 and NQO-1 of Nrf2, thereby limiting APAP-induced liver toxicity (22). Experimental studies have shown that CGA protects against CCl4-induced elevation of serum transaminase and abnormal liver pathology, which also achieves the purpose of protection by enhancing the anti-oxidant pathway mediated by Nrf2 (82). CGA can also ease kidney damage induced by the imbalance of the oxidative system. The causes of renal ischemia/reperfusion (I/R) injury include partial resection of kidney tissue, kidney transplantation, iatrogenic trauma, sepsis, and shock, etc., which often leads to oxidative stress. Studies have revealed that CGA uses a certain amount of its own R-OH free radicals to form hydrogen free radicals, neutralize ROS generated by oxidative stress, and target I/R (83).

Additionally, CGA can also achieve liver and kidney protection by controlling cell apoptosis. Accumulation of saturated fatty acids can readily cause liver cell apoptosis and endoplasmic reticulum stress, which can be the fuse of hepatitis, liver fibrosis, cirrhosis, and even liver cancer. CGA can limit increased levels of the endoplasmic reticulum stress markers—glucose regulated protein 78 (GRP78), C/EBP homologous protein (CHOP), and glucose regulated protein 94 (GRP94), prevent palmitic acid-mediated hepatocyte apoptosis (23). Tamoxifen (TAM) is used as the gold standard for treating early and advanced breast cancer, but the damage of TAM to liver and kidney function cannot be ignored. The experimental results of comparing CGA combined with TAM and TAM alone to treat breast cancer showed that the TAM group acting alone significantly increased the activity of Caspase-9 and Caspase-3 in liver and kidney tissue, while 25 and 50 (mg/kg) CGA combined with TAM groups, the above indicators were reduced, and 50-mg/kg was significantly better than 25-mg/kg, which means that CGA may protect liver and kidney tissue from apoptosis in a dose-dependent manner via the external or internal pathways of apoptosis (24). The development of kidney disease is similar to that of the liver. CGA is also involved in the regulation of cell apoptosis and slows down the development of kidney disease. Renal fibrosis is mainly mediated by transforming the growth factor-β1 (TGF-β1) and bone morphogenetic protein-7 (BMP-7). Hepatocyte growth factor (HGF) has mitotic, anti-fibrotic, and anti-apoptotic effects on kidney epithelial cells. Death activity can promote regeneration and restrict kidney fibrosis initiation and progression. HGF restricts the expression of TGF-β1 in renal fibrosis, and inhibit podocyte apoptosis and endothelial cell apoptosis through the cellular mechanism of Smad2 and Smad3 in TGF-β1, while inhibiting tubular epithelial cell apoptosis and epithelial-mesenchyme transformation, and activation of mesenchymal fibroblasts. CGA regulates the expression of HGF and protects from renal fibrosis, but the process of CGA regulation of HGF needs to be further studied to obtain accurate results (25).

Acute hepatitis, including viral hepatitis and autoimmune hepatitis, is a significant threat to human health globally. CGA can resist inflammation and slow down the development of liver diseases. In the established concanavalin A (ConA)-induced hepatitis model, experiments confirmed that CGA can reduce the infiltration of macrophages, neutrophils, and activated CD4 T lymphocytes into the liver, but also reduce the exposure of ConA. The production of mouse serum pro-inflammatory cytokines tumor necrosis factor-α (*TNF-*α) and interferon-γ (*IFN-*γ), indicates that CGA restricts the infiltration of white blood cells and the production of pro-inflammatory cytokines to inhibit the occurrence of hepatitis. Other results indicate that Toll-like receptor 4 (TLR4) plays a negative role in the inflammatory response. Interleukin-1 receptor-associated kinase 1(*IRAK-1*) is a key downstream molecule of the TLR4 pathway, and its phosphorylation can activate a series of inflammatory pathways (84, 85), but in experiments, it was detected that CGA down-regulated the expression of TLR4 in the liver of mice exposed to ConA, and inhibited *IRAK-1*, NF-κB, and mitogen-activated protein kinase (*MAPK*). Activation, indicating that the hepatoprotective effect of CGA may be obtained by down-regulating TLR4 signal activation (26). CGA can also inhibit inflammation, thereby building a healthy barrier to kidney = damage induced by long-term exposure to heavy metals. Lead (Pb) exposure is an environmental problem globally, and its exposure can lead to severe kidney damage. Experimental studies have shown that long-term exposure to lead can alter the structure of the renal tubular epithelium and enlarge the tubular epithelium, leading to a series of chronic or acute nephrotoxic reactions, and CGA can inhibit the activation of the NF-κB signaling pathway. Blocking the NF-κB signaling pathway increases the gene expression of pro-inflammatory factors *TNF-*α, interleukin 1β (*IL-1*β), and interleukin 6(*IL-6*) (27). Some researchers have also discovered that for long-term use of vancomycin (VCM), resulting in mitochondrial damage, kidney reabsorption of energy-dependent components is affected and induces nephrotoxicity. CGA can reverse VCM-induced nuclear transcription factor NF-κB, induce the expression of nitric oxide synthase (iNOS), and downstream pro-inflammatory mediators *TNF-*α, *IL-1*β, and *IL-6* (28). The significant protective effect of CGA on liver and kidney injury may be related to its antioxidant and anti-inflammatory activities. Collectively, there is strong evidence that regulation of various pathways by CGA suppresses the production of inflammatory cytokines and oxidative damage, leading to reduced liver fibrosis and carcinogenesis. It provides a new solution for the accumulation of liver and kidney toxicity of exogenous chemicals *in vivo*.

### Anti-bacterial

**Figure 6:**
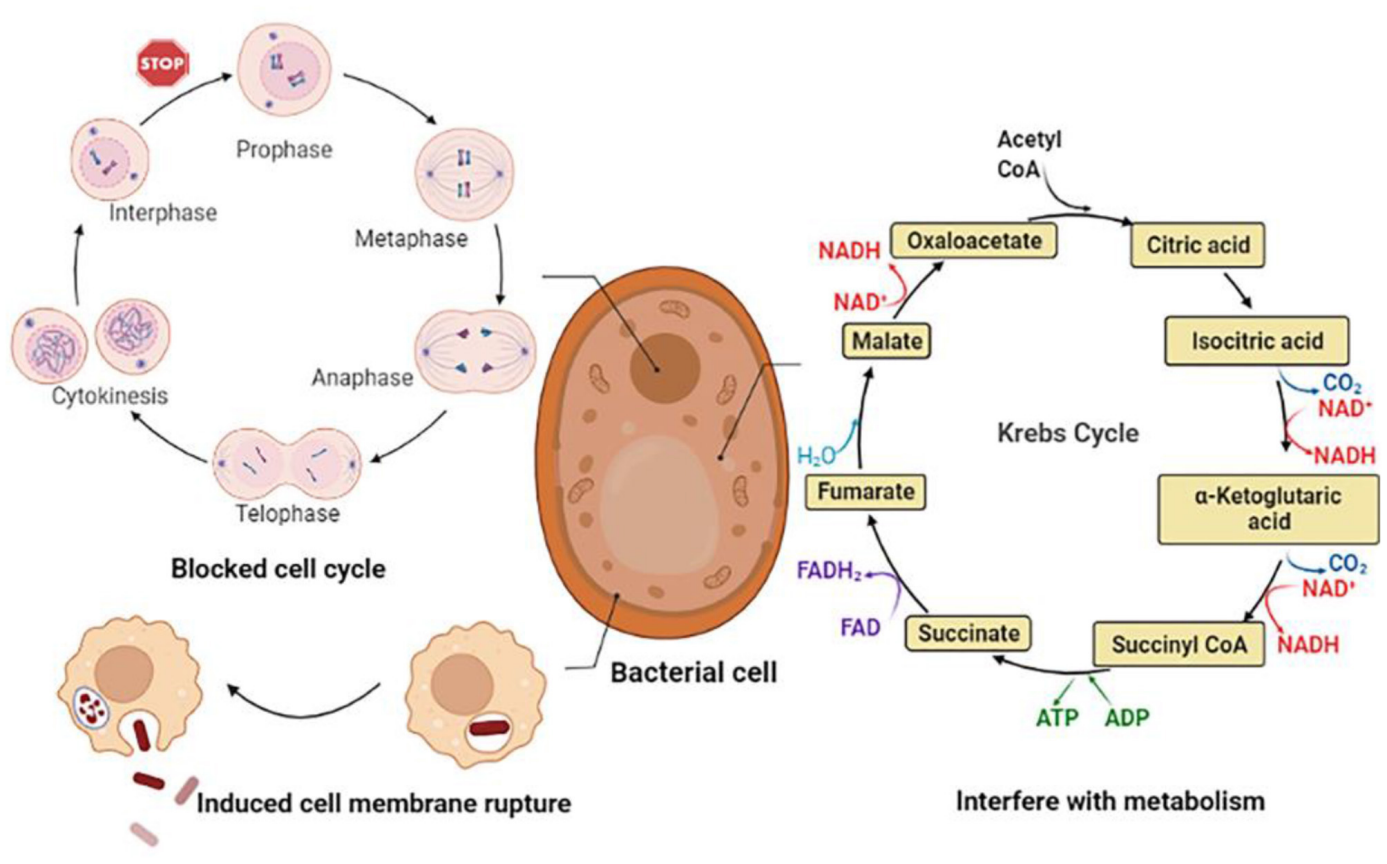
Bacteriostatic mechanism of CGA.

CGA has a better inhibitory effect on both Gram-positive and negative bacteria. The main mechanisms can be summarized as follows: (1) Destroy the structure of cell membranes, causing leakage of intracellular metabolites and trigger cell inactivation; (2) Interfering with normal cell cycle progression, thereby inhibiting the growth of microorganisms; (3) Disturb the normal metabolic activities of bacterial cells, leading to metabolic disorders within the cells. The anti-bacterial mechanism is shown in Figure 6.

CGA achieves the purpose of bacteriostasis by inhibiting the synthesis of bacterial cell membranes, resulting in the loss of cell contents and bacterial inactivation. It had been reported that CGA had a good inhibitory effect on the food-borne pathogen *Pseudomonas aeruginosa P1* and the degree of inhibition increases with increasing concentration: 0.125% CGA partially delayed *P. aeruginosa P1*; 0.25% CGA could partially inhibit the growth of *P. aeruginosa P1*; 0.5% CGA could completely inhibit the growth of *P. aeruginosa P1*. while altering the expression of lipopolysaccharide (LPS) biosynthesis genes derived from the major component of the outer membrane of *P. aeruginosa P1*. CGA induced the detachment of LPS, resulting in enhanced cell membrane permeability, cell membrane depolarization, leakage of intracellular proteins and ATP, disturbance of cell metabolism and ultimately cell death (29). The antibacterial mechanisms of CGA on the food-borne pathogen *Salmonella enteritidis S1* in chilled chickensconsisted two aspects. On the one hand, destroying the structure of the outer membrane (OM), inner membrane (IM) and cell wall of *S. enteritidis S1* could lead to the leakage of cell contents; on the other hand, CGA hindered the main energy metabolism enzyme succinate dehydrogenase (SDH) and malate dehydrogenase (MDH) activity. The data showed that after 1/2 MIC and 1 MIC CGA treatment, MDH activity decreased from 0.823 to 0.402 and 0.295 U/mg, and SDH activity decreased from 38.25 to 19.36 and 15.23 U/mg. It was suggested that CGA could play a part in inhibiting bacterial growth by interfering with the energy metabolism of *S. enteritidis S1*. The two aspects worked together to promote cell death (30).

To study the effect of CGA combined with levofloxacin (LFX) on *Klebsiella pneumoniae* (KPN) biofilm formation *in vitro*, the study found that 512 μg/mL CGA could effectively inhibit the formation of KPN extended-spectrum β-lactamases (ESBLs) biofilm, and combined with LFX could achieve better synergistic effect. Fractional inhibitory concentration index (FICI) was <0.5 in 3 strong ESBLs biofilm-positive strains (31). For bovine mastitis caused by *Staphylococcus aureus* and *Escherichia coli*, CGA appears to be a better alternative strategy than antimicrobials that are highly susceptible to drug resistance. Studies had shown that 10, 20, and 30 μg/mL CGA had no cytotoxic effect on bovine mammary epithelial cells (BMEC) in culture; 20 μg/mL CGA enhanced the viability of BMEC exposed to *S. aureus*; 30 CGA at μg/mL reduced *S. aureus* growth after 9 h. *S. aureus* biofilm formation also tended to be reduced with the addition of 10, 20, and 30 μg/mL of CGA compared to the untreated *S. aureus* group (86).

CGA blocks the normal progress of the cell cycle, thereby affecting cell growth. Visceral leishmaniasis caused by Leishmania, commonly known as kala-azar, is the second-largest parasitic disease after malaria. Some researchers have studied the limiting effect of CGA on Leishmania. After 48 h of CGA treatment, the protoflagella of Leishmania changed into a spherical shape, the flagella disappeared, the exercise capacity decreased, the metabolic activity decreased, and the growth reversed. In the logarithmic phase, the researchers discovered that the growth of flagella at the G1/S checkpoint was significantly blocked (87). *Fusarium* is one of the causes of rot and spoilage of fruits and vegetables. Studies have shown that CGA triggers the outbreak of ROS in *Fusarium vine* niger hyphae, induces bacterial cell apoptosis, and has a considerable inhibitory effect on conidial germination, germ tube elongation, cell viability, and hyphal growth of *Fusarium vine* niger. So we believe that the use of CGA may effectively prevent damage to fruits and vegetables after picking, and serve a vital role in the fruit and vegetable industry (88).

CGA interferes with the normal metabolism of bacteria, triggers cell metabolism disorders, and achieves anti-bacterial effects. Quorum sensing (QS) is a communication between cells that bacteria use to regulate collective behavior. QS controls the production of virulence factors of many bacterial species, and experiments have shown that after CGA treatment, the expression of QS-related genes is significantly down-regulated. It is believed that the mechanism of action may be that CGA forms hydrogen bonds with three QS receptors to restrict the function of QS and reverse the virulence regulation of bacteria (32). *Bacillus subtilis* has a strong ability to hydrolyze starch and protein, and this microorganism usually causes food spoilage. The experimental results show that although CGA cannot directly destroy the cell membrane of *Bacillus subtilis*, it can cause a significant decrease in the intracellular adenosine triphosphate (ATP) concentration. Metabolomics results show that CGA can cause TCA and glycolysis by inducing the TCA cycle (TCA) and glycolysis. This results in intracellular metabolic disorder to achieve anti-bacterial effect (33).

### Anti-tumor

**Figure 7:**
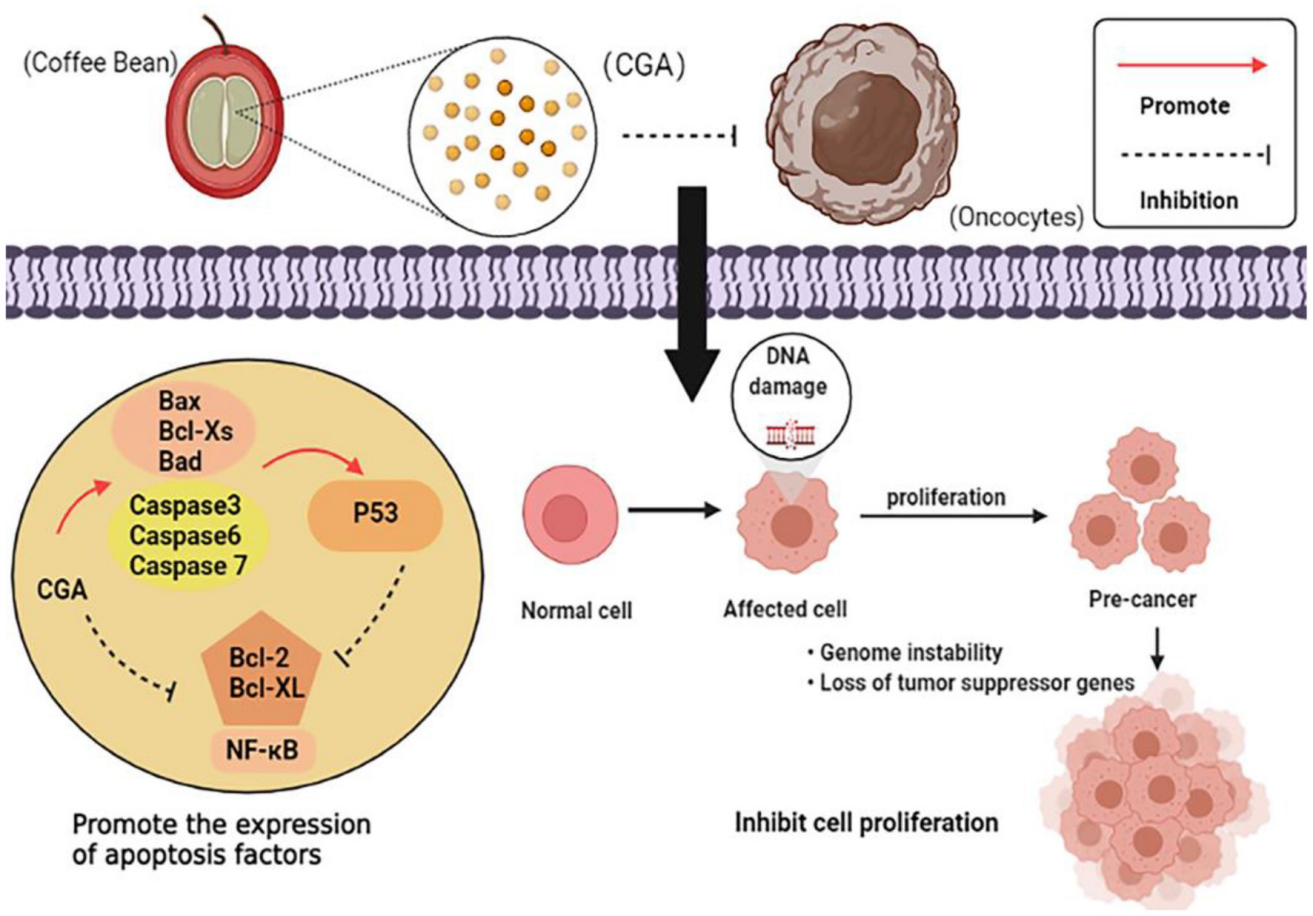
Anti-tumor mechanism of CGA.

CGA plays a major role in the prevention and inhibition of tumor growth, and the anti-tumor mechanism can be summarized as follows: (1) Regulate the expression of apoptosis-related factors and promote apoptosis of cancer cells; (2) It acts on the cell division cycle and hinders the reproduction, metastasis, and invasion of cancer cells; (3) Affect the metabolism level of cancer cells and the normal growth of cancer cells. The anti-tumor mechanism is shown in Figure 7.

Currently, one of the most effective therapies for cancer is targeting the apoptosis pathway. Polymerase chain reaction showed that CGA could down-regulate the expression of anti-apoptotic genes *Bcl-2* and *Bcl-XL*, as well as up-regulate the expression of pro-apoptotic genes *Bax, Bcl-XS*, and Bad (34, 35). It also induces the expression of p38 mitogen-activated protein kinase (p38 *MAPK*) and c-Jun amino-terminal kinase (*JNK*) to promote apoptosis of cancer cells (36). Experiments have confirmed the combined anti-cancer effects of CGA with the major microbial metabolites of colon caffeic acid (CA), 3-phenylpropionic acid (3-PPA), and benzoic acid (BA). 3-PPA significantly reduces the ratio of mitochondrial DNA (mtDNA) to nuclear DNA (nucDNA) and effectively induces cell apoptosis during tumorigenesis (89). *P53*, as a multifunctional protein, regulates *Bax* and *Bcl-2* gene expression, apoptotic response, and DNA repair mechanisms to maintain genomic stability, while CGA upregulates *P53* gene expression and indirectly regulates apoptosis of cancer cells (37).

CGA also inhibits the proliferation, migration, and invasion of cancer cells. CGA synergizes the inhibition and metastasis of cancer cell growth mediated by Regorafenib. The combination of Regorafenib and CGA blocks the progression of the cell cycle from S, to G2/M and restricts the division of cancer cells (34). This has also been noticed in human colon cancer cells, where CGA and its metabolites hinder the cell cycle at G0/G1, S or G2/M, and cell proliferation is restricted due to the cycle blockage (89). Additionally, CGA blocking the binding of membrane-linked protein A2 (ANXA2) to actin may also block cell cycle progression in the G0/G1 phase, thereby inhibiting tumor cell cycle and migration (90). Jiang's group studied the induction effect of CGA on various solid tumor lines (human liver cancer Hun7 cells, lung cancer H446 cells). Both 25 and 50 μM CGA treatment observed that tumor cell growth was inhibited and migration was inhibited. CGA induces solid tumor differentiation and inhibits cancer cell proliferation, migration, invasion, mitochondrial ATP production, and tumor clone formation. CGA increases SUMO1 protein expression by stabilizing SUMO1 mRNA, which in turn downregulates expression of miR-17 family members (miR-20a, miR-93, and miR-106b). Decreased expression of three miR-17 family members promotes p21 expression, leading to cell differentiation. CGA activation of the c-Myc threoylation/reduced miR-17/elevated p21 axis leads to cell differentiation and attenuates cancer behavior in solid tumor cells (38). Cancer stem cells play an essential role in the mechanism of drug resistance and tumor recurrence of cancer cells. Data studies have indicated that CGA reduces the expression of self-renewal related stem cell marker genes *Nanog, POU5F1, Sox2, CD44*, and *Oct4*, and inhibits the migration of cancer cells (36).

CGA controls the metabolic response of cancer cells and disrupts normal cell growth. Melanoma is most commonly found in skin cancer and often causes death associated with skin cancer. Static magnetic fields (SMF) and bioactive compounds found in food are potential for cancer treatment. Some scholars have studied the effect of SMF induced by neodymium magnets on CGA-treated melanoma cells, and found that when CGA-treated cells were placed in 0.7 T magnetic field, the anti-oxidant enzyme system of cancer cells was significantly inhibited, the activities of SOD, *GSH-Px*, and CAT in cell lysis fluid decreased, and the anti-oxidant system was disabled. Rapid proliferation of ROS results in a large amount of cell damage, leading to cell death (91). Additionally, the combination of 250-mmol/L CGA and 20-mmol/L fluorouracil-(5-FU) was shown to promote the rapid proliferation of ROS, inhibit the activation of *MAPK*/*ERK*, and inhibit the proliferation of cancer cells (39). Controversially, a large number of literatures have reported the positive effect and mechanism of CGA on anti-tumor, but the views are not consistent. Therefore, the search for potential anti-tumor targets and the mechanism of action need to be further explored.

### Regulation of Carbohydrate and Lipid Metabolism

The intake of high-carbohydrate and high-fat diet often results in the secretion of lipopolysaccharide throughout the body, and increases the content of chylomicrum in the intestinal tract, leading to metabolic endotoxemia and inflammation, which are the development basis of obesity, hypertension, and metabolic abnormalities. The mechanism of CGA in regulating glucose metabolism and lipid metabolism can be summarized as follows: (1) CGA acts on the expression of enzymes and genes related to glucose metabolism improves insulin sensitivity, thus, alleviates the development of diabetes; (2) Act on genes and proteins related to fatty acid metabolism to reduce fatty acid deposition.

CGA regulates the expression of enzymes and genes related to the glucose metabolic pathway, increases insulin sensitivity, and reduces glucose accumulation to inhibit the further development of diabetes. Hyperglycemia can cause oxidative stress to produce many ROS, reduce the expression of anti-oxidant enzymes such as CuZnSOD (*SOD1*) and MnSOD (*SOD*2), increase AGEs, activate protein kinase C (PKC) dependent *ERK* signaling pathway, and cause liver dysfunction. CGA was found to reverse these indices and have a beneficial effect on diabetes (40–42). Other studies have shown that CGA can decrease fasting blood glucose (FPG) level and promote insulin secretion in patients with impaired glucose tolerance to alleviate diabetes (43). CGA itself does not affect phosphorylation of phosphatidylinositol-3-hydroxykinase (PI3K) and insulin receptor substrate (IRS-1), but metabolites of CGA can cause the phosphorylation of PI3K and IRS-1, thereby enhancing insulin sensitivity and playing a positive role in treating type 2 diabetes (44). Additionally, CGA formed a complex (1:1 molar) with α-glucosidase (α-GLU) and α-amylase to exert anti-hyperglycemic effects *in vitro* (45). With acarbose as the positive control, the enzyme inhibitory potential of CGA was measured by the half maximal inhibitory concentration (IC50). The experimental results showed that the IC50 values of acarbose for α-amylase and α-glucosidase were 16.45 and 23.36 μg/ml, respectively. While the IC50 values of CGA for α-amylase and α-glucosidase were 21.93 and 27.14 μg/ml, respectively, these data show the great potential of CGA in anti-diabetes (92). Furthermore, CGA promotes glucose uptake by upregulating glucose transporter 2 (*GLUT2*) and phosphofructokinase (PFK), which in turn stimulates the oxidative phosphorylation of brown adipose tissue (BAT) and adenosine triphosphate, activates the thermogenesis of BAT, and promotes its release of fibroblast growth factor-2 (FGF2) endocrine. Improving lipid metabolism may also be a potential therapeutic approach for metabolic diseases (46, 47).

CGA regulates the expression of proteins and enzymes related to fatty acid metabolism and reduces the fatty acid deposition. Obesity is a global health problem related to various metabolic diseases, including diabetes, cardiovascular disease, stroke, some cancers, and nonalcoholic liver disease (NAFLD) (93, 94). Some researchers have attempted to evaluate the effects of polyphenol-rich herbal compounds (hawthorn/ellagic acid, CGA, cinnamon/cinnamic acid) on obesity in a high-fat diet induced obesity mouse model. The combination of herbal preparations significantly increased serum high-density lipoprotein (HDL) levels. Decrease deposition of fat droplets in the liver, and intima media thickness in the aorta. It also increased the activity of carnitine palmityl transferase (CPT) and significantly decreased the activity of β-hydroxylβ-methylglutamyl-coA reductase and fatty acid synthase (FAS) (48), effectively protecting metabolic dysfunction. Studies have revealed that the combined action of CGA and caffeine can help prevent obesity, as well as metabolic and lifestyle-related diseases through the AMPKα-LXRα/SrebP-1C pathway. AMPK is an essential cellular energy sensor that plays a crucial role in regulating fatty acid metabolism and energy homeostasis (95).

The combination of CGA and caffeine significantly promoted the phosphorylation of AMPKα, upregulated hormone-sensitive lipase (HSL) expression, and adipotriglyceride lipase (ATGL), inhibited the expression of transcriptional regulatory factors (SREBP-1C and LXRα), and decreased the expression of FAS. Because triglycerides (TG) are broken down into large amounts of free fatty acids, which can stimulate liver inflammation and worsen hepatocyte insulin resistance, the combined use of CGA and caffeine significantly increases ACO expression, a key rate-limiting enzyme involved in liver fatty acidβoxidation, contributing to fatty acid decomposition and energy usage (49). Related experimental studies have also shown that CGA can decrease the excess fat deposition by reducing the mRNA expression of FAS, acetyl-CoA carboxylase, and stearoyl-coA desaturase in liver (50).

### Anti-inflammatory

CGA directly acts on the NF-κB signaling pathway to control the expression of pro-inflammatory and anti-inflammatory factors. The NF-κB signaling pathway is a crucial key to controlling pro-inflammatory and anti-inflammatory factors. It consists of structurally related proteins p50, p52, p65, c-RelA, and RelB, and is called the Rel family. The activation of NF-κB is mediated through phosphorylation of its limiting subunit IκB kinase complex. IκB is degraded after phosphorylation and releases the subunit p65 of NF-κB, which transfers to the nucleus and interacts with the regulation of anti-inflammation, combining with specific gene promoters of pro-inflammatory proteins to initiate an inflammatory response. The anti-inflammatory and pro-inflammatory activities of NF-κB depend on its physiological environment (51). The human intestinal Caco-2 cell line is widely used in the intestinal barrier model. Some researchers have studied the protection of CGA isoforms on the inflammatory response of *IFN-*γ and myristate (PMA) mixed stimulated differentiation of Caco-2 cells effect. After Caco-2 cells ingest CGA isomers, due to the anti-oxidant properties of CGA when it reacts with free radicals, it may produce a low-level pro-oxidant that promotes the activation of NF-κB, which makes NF-κB anti-inflammation active (96).

CGA regulates the expression of proteins and genes in the inflammatory response to hinder various damages caused by inflammation to the body. HO-1 is an important downstream protease regulated by Nrf2. Related experiments have confirmed that HO-1 effectively inhibits IkB phosphorylation and its subsequent degradation, leading to NF-κB translocation and downstream inflammatory gene transcription reduction, which indicates that CGA activates Nrf2/HO-1 and inhibit NF-κB (52). Other studies have also shown that mice that knock out the IL-10 gene are a good model of inflammatory bowel disease. In the absence of IL-10, inflammatory infiltrates (including lymphocytes, plasma, etc.) will occur in mice. The accumulation of cells, macrophages, eosinophils, and neutrophils), and increased expression of inflammatory proteins such as iNOS and cyclooxygenase-2 (COX-2), and rapid immunoglobulin E(IgE) expression, the steady-state ratio 2 of helper T cells (CD4+) to cytotoxic T cells (CD8+) also changed. After CGA treatment, the above indicators were effectively improved (53). Weaned piglets can also cause intestinal barrier inflammation due to weaning. In the mechanism study, it is found that the TLR4/NF-κB signaling pathway may play a vital role. TLR4 is a typical member of the TLR family and is widely distributed in various intestinal cells (97). Activated TLR4 can stimulate the activation of the NF-κB signaling pathway, thereby increasing the expression of *IL-6, TNF-*α, *IL-1*β (98), and other inflammatory factor genes. After adding CGA to the feed, the mRNA expression levels of TLR4 and its downstream signals (including IRAK1 and TRAF6) were significantly down-regulated, while the negative regulatory factors related to the TLR4 signaling pathway, including Tollip, RP105, and SOCS1, were significantly increased (54). Additionally, in the inflammation of spinal cord injury rats, CGA has also been recognized to down-regulate *TNF-*α and IL-Concentration levels of 1β and *IL-6* (55).

### Protect the Nervous System

Autophagy and apoptosis are two different types of programmed cell death, which play an essential role in the development and maintenance of homeostasis in multicellular organisms. Autophagy is one of the cytoprotective mechanisms by which excessive or damaged organelles are degraded. The purpose of apoptosis is to eliminate dead cells during cell proliferation or differentiation. More studies have shown that autophagy and apoptosis are involved in nervous system damage (99). Some researchers have discovered that CGA has a protective effect on corticosteroid-induced neurotoxicity of PC12 cells, which is reflected in the fact that CGA can enhance neuronal cell viability, inhibit the transformation process of autophagosome marker LC3-I to LC3-II, and reverse the decreased Akt/mammalian target of rapamycin (mTOR) pathway activity. These phenomena show the positive side of CGA in treating depression and pave the way for the research and development (R & D) of anti-depressants (56). Additionally, CGA may play a positive role in spatial memory impairment and hippocampal neuronal and vascular response defects after transient global cerebral ischemia by increasing mRNA expression of apoptosis regulator *Bcl2*, anti-oxidant enzyme *SOD2*, and endocortisol-marker CD31, and decreasing mRNA expression of vasoconstrictor (ET-1). Improving the apoptosis of endothelial cells, thereby protecting transient memory loss (57, 58).

A large amount of evidence shows that removing soluble amyloid-β (Aβ) peptide deposits help prevent the progression of Alzheimer's disease (AD) (100). Transcription factor EB (TFEB), a basic helix-loop-helix leucine zipper transcription factor controlled autophagy-lysosome pathway, is believed to play a vital role in clearing Aβ. Existing analysis has dicorvered that CGA can significantly increase the expression of cathepsin D (an aspartic protease that is important for lysosomal proteolysis) and inhibit the mTOR pathway to improve the translocation of TFEB to the nucleus. Thereby enhancing the lysosomal activity of cells and protecting neuronal damage (59). Another study found that local administration *in vivo* can attenuate the activity of rat trigeminal caudate nucleus neurons (SpVc) induced by mechanical stimulation. Subcutaneous local injection of CGA may inhibit the excitability of SpVc neurons in response to harmful mechanical stimulation by activating the voltage-gated potassium channels of the nociceptive nerve terminals of the trigeminal ganglion neurons and regulating acid-sensitive ion channel ASICs. The average frequency of CGA's suppression of SpVc neuron discharge can be compared with local anesthetics, sodium channel blockers, and 1% lidocaine, and the efficacy is similar. Therefore, CGA is expected to become a new generation of natural plant extracts for treating trigeminal neuralgia (101). Despite the increasing evidence showing that long-term coffee intake, high in CGA can attenuate neurodegeneration, the molecular mechanisms of the protective effects of CGA against neurodegeneration are complex and thus additional, formal, well-controlled preclinical and clinical studies are needed to elucidate such mechanisms.

### Action on Blood Vessels

In recent years, several studies on animal models of hypertension have highlighted the potential antihypertensive effects of CGA. For the exploration of its mechanism, Esther et al. found that CGA had a direct vasodilatory effect through an endothelium-dependent effect, and the main pathways involved include nitric oxide synthase (NOS), cyclooxygenase (COX), and endothelium-derived supernatant. Polarization Factor (EDHF). For acetylcholine (ACh)-induced vasodilation, CGA exhibited duality, with CGA at low concentrations (10^−6^ mol/l) potentiating ACh-induced relaxation and reducing norepinephrine contraction; while high concentrations (10^−4^ mol/l) significantly inhibited ACh-related relaxation. The new pharmacological effects of CGA for vasodilation can propose new treatment methods in hypertensive diseases, but the dosage needs to be paid attention to (102). In an experimental study of healthy volunteers, acute ingestion of 400 mg CGA (equivalent to two cups of coffee) resulted in a significant decrease in systolic blood pressure (SBP) and diastolic blood pressure (DBP), by 2.41 and 1.53 mmHg, respectively. It will benefit cardiovascular health if sustained (103).

Furthermore, CGA was able to protect isolated mouse aortic rings from hemin (HOCl)-induced endothelial dysfunction and enhance endothelial cell survival after HOCl-induced oxidative damage. And CGA increased endothelial NO production in a dose-dependent manner, 5 and 10 μM CGA increased eNOS dimerization at 6 h, and induced HO-1 at 6 h and 24 h, which plays an active role in vascular protection. These all indicated that CGA could exert beneficial effects on blood vessels through eNOS/NO pathway and oxidative stress/HO-1 pathway (104). These results provide further evidence for the vascular protective effect of dietary polyphenols.

## Application of CGA in Food

### Food Additives

#### Emulsifier

Emulsification, aggregation, flocculation, and other undesirable phenomena often occur in emulsion systems during storage. Generally, protein hydrolysates are selected as an emulsifying stabilizer to improve the resistance of products. Due to the poor distribution of protein hydrolysates at the oil-water interface, their popularity is limited. Phenolic compounds are easy to combine with proteins, so adding phenolic oxidants into oil-in-water protein emulsions to improve stability has become the focus of many scholars. It has been reported that CGA is oxidized to quinone by polyphenol oxidase under alkaline conditions, and dimers are produced in the side reaction. The protein hydrolysate forms covalent C-N or C-S bonds with the phenol ring, and its amino or sulfhydryl side chain may also react with the dimer to cause cross-linking of the protein hydrolysate and improve the physical stability of the film around the oil droplet. Simultenously, CGA can inhibit the generation of free radicals in the lipid oxidation process and restrain the development of lipid oxidation, so that the modified emulsion has high oxidation stability (105). In food processing and storage, the molecular conformation, aggregation, and functional properties of proteins are also easily damaged. Polyphenols can inhibit oxidation and protect the interface structure of the emulsion. Therefore, some researchers try enhancing the functional properties of proteins by combining polyphenols and polysaccharides. By establishing a protein-chlorogenic acid-dextran conjugate (PCD) model, exposed internal protein amino acid residues in the polar environment, reduced the interfacial tension, and enhanced anti-oxidant activity in different degree. Compared with individual protein emulsions, PCD coupling has the smallest particle size and the best stability. PCD coupling has a good developmental direction in food emulsifiers (106).

#### Colorant

Anthocyanins are increasingly used to replace synthetic pigments. However, their color is extremely sensitive to food processing that requires heating. Copigmentation between CGA and quercetin and Mulberry anthocyanin (AC) occurred through intermolecular and intramolecular interactions, which prevented the nucleophilic attack of anthocyanin xanthyl cation and stabilized the color of anthocyanin. This is due to stronger binding occurred between multiple ligands and AC than single ones due to their extra -OH, -COOH groups, and delocalization systems. The binding was allowed by increased H-bonding, van der Waals forces, and π-π sites by the extra groups of the multiple co-pigments with AC in aqueous juice and whey particle-based models. Therefore, studies on terpolymer mixtures of phenolic acid-flavonol-anthocyanin are expected to be a promising food colorant (107). A red pigment can be produced by oxidative coupling of CGA and tryptophan (TRP) in air at pH 9. CGA-TRP pigment shows satisfactory water solubility and extinction coefficient in a wide pH range (1–12) and temperature up to 90°C, which is 5–20 times higher than the commonly used natural red dye preparations. Doses as low as 0.01% are permitted. It provides a natural red color to milk and fatty foods, and has the advantage of being safer and cheaper than synthetic azo pigments that are still in use and have serious toxicity. Thus, it is expected to become a new generation of pigment replacement (108).

#### Preservative

CGA causes cell death by inducing changes in the permeability of S1, OM, and IM of food-borne pathogens, releasing intracellular proteins and ATP, inhibiting the activity of SDH and MDH, disrupting bacterial metabolism. Based on the anti-bacterial effect of CGA, it is predicted to be used as a new food preservative in food production (30).

### Extend Food Storage Time

Enzymatic reactions are related to lipid oxidation during transportation, processing, and storage of aquatic products and their products. Endogenous lipase and lipoxygenase play an essential role in promoting lipid oxidation. CGA can limit lipid oxidation of grass carp muscle during cold storage. The specific mechanisms of action lie in the formation of hydrogen bonds between CGA and lipase, hydrophobic interactions between tryptophan (Trp) near the benzene ring and acyl binding bag, and weak interactions, such as van der Waals forces with surrounding amino acid residues, which together competitively inhibit the binding sites of endogenous lipase and lipoxygenase (LOX) (109). The sword shrimp is rich in protein and fatty acids, especially docosahexaenoic acid (DHA) and eicosapentaenoic acid (EPA). However, in the process of capture, processing, storage, distribution, and commercialization of prawn, it is easy to be invaded by bacteria. Under the action of endogenous enzymes, the protein in prawn meat is easily decomposed, resulting in black spots or melanosis on the surface. Based on the inhibitory effects of CGA on microbial growth, lipid oxidation, and protein degradation, some researchers studied the pH value, Ca^2+^-ATP activity, and total viable bacterial count of shrimp during cryopreservation using chlorogenic acid-gelatin (CGA-Gel) combined with cryopreservation at −5°C. The results indicated that the pH value of shrimp meat was significantly lower than that of the control group after CGA-GEL combined treatment, and the decrease in Ca^2+^-ATP activity and the proliferation of spoilage bacteria were delayed, and the decay of shrimp was effectively delayed (110).

Sea cucumber is favored by the public because of its high concentration of protein, vitamins, and minerals, as well as bioactive substances, such as polyphenols, triterpenoid glycosides, and free radical scavengers. However, fresh sea cucumber is prone to autolysis because of the influence of enzyme, microorganism, and many environmental factors. Traditional practices will cause serious loss of some nutrients of sea cucumber, affecting taste and edibility; the ready-to-eat sea cucumber treated using high pressure steam is also easy to degrade during storage. These disadvantages have seriously affected the quality and economic value of sea cucumber. Some scholars have examined celery from natural fruit and vegetable juice to protect the sea cucumber's taste, color, and quality. It was found that CGA with the most abundant content in celery juice played a significant role in the phenol hydroxyl by covalent or hydrogen bonds that CGA and collagen polarity side chains of amino acid residues-NH2 groups, through covalent bonding or hydrogen bonding. Thus, the water migration and fracture of collagen fibers in sea cucumber and collagen degradation during storage improved (111).

CGA is the primary phenolic acid in soy milk and has the highest concentration of all phenolic acids in soy products. Studies have reported that soy milk rich in fish oil exhibits higher lipid oxidation stability than milk, and the significantly reduced concentration of CGA in soy milk storage suggests that this compound may contribute to the oxidative stability of soy milk rich in fish oil. However, further studies are needed to confirm the specific anti-oxidant mechanism of CGA (112).

### Food Ingredient Modification

The polyhydroxyl structure of CGA has also been applied in protein modification. Whey protein isolate (WPI) is an important food component, and the main proteins in WPI, β-lactoglobulin (βLG), and α-lactalbumin (αLA), including bovine serum albumin, are potential allergens. Traditional methods to eliminate allergens have low sensory evaluation and are susceptible to light, oxygen, and temperature. Interactions between dietary polyphenols and proteins have been shown to decrease the allergenicity of specific food allergens. When the CGA and WPI coupling reaction occur, conjugate formation of covalent bonds, the covalent interaction between the two led to the free amino and free thiol group content reduction, the structure of the protein expands, WPI conformational change, thus affecting the IgE linear table, and reduces the IgE combining ability of WPI. The digestibility, solubility, emulsifying activity, foaming property, and anti-oxidant capacity of WPI were improved (113–116).

Additionally, CGA covalently binds to Ara h1, the most abundant allergen in peanuts. By reducing the content of α-helix and increasing the content of random helices, the folding structure and secondary structure of the protein are increased, and the binding ability of Ara h1 and IgE is significantly decreased. Improving the digestibility of pepsin, and better solve the problem of people's allergy to peanuts (117). Furthermore, lutein exists in marigold flowers and has been used as a natural fat-soluble pigment for a long time, but its poor water solubility, poor chemical stability, and low bioavailability limit its wide application in food. The emulsion system stabilized by the terconjugate of chlorogenic acid (CA), WPI, dextran (DEX), and added with vitamin E (VE) has been proved to enhance the chemical stability of lutein. To address the limitations of lutein in the food industry due to poor water solubility, chemical instability, and low bioavailability (118).

Lotus seeds, are considered a top-notch tonic, starch is the main ingredient of lotus seeds, accounting for 60% of the total dry weight of lotus seeds. Additionally, it also contains many phenolic components, such as CGA, which is primarily found in lotus embryos. In the original state, since the molecules of amylose and amylopectin are completely accumulated, starch granules are difficult to digest and absorb. To meet the needs of consumers and producers, the modification of starch has become a research focus of many food workers. Microwave (MW) radiation, as a non-ionizing radiation energy, can effectively change the structure and functional properties of food materials. The treatment of MW caused more CGA in lotus seeds to migrate to the starch. CGA not only occupied the active sites of digestive enzymes (α-amylase and amyl glucosidase), and gradually reduced the catalytic efficiency; Starch forms a complex because the complex cannot form a new periodic molecular organization due to the irregular spiral arrangement, which acts as a diffusion barrier to enzymes and water, thereby delaying hydrolysis. In summary, lotus seeds treated with MW are of great value for developing high starch foods with slow digestion characteristics (119, 120).

### Food Packaging Materials

Recently, traditional food packaging materials using petrochemical plastics as raw materials have brought food safety and environmental challenges. Biodegradable bio-based food packaging materials, such as polysaccharides, proteins, lipids, etc, have attracted considerable attention. Chitosan (CS) is a linear natural cationic polysaccharide derived from the deacetylation of chitin. Because of its non-toxic, biodegradable, biocompatibility, and inherent anti-bacterial properties (121), the CS film has low anti-oxidant and anti-bacterial activities (122), which inhibits its application. Improving the performance of CS film by adding natural anti-oxidants and anti-bacterial substances is a trending research topic presently. To explore the physical, mechanical, and biological properties of CA-g-CS conjugated membrane and CA-CS composite membrane, the researchers prepared CA-g-CS conjugated membranes (CA-g-CS) with the same amount of chlorogenic acid. I, CA-g-CS II, and CA-g-CS III) and CA-CS composite membranes (CA-CS I, CA-CS II, and CA-CS III), and analyzed the two kinds of membranes during storage of shrimp with the effect of weight loss rate, pH, total volatile basic nitrogen, and the total number of bacteria. According to the experimental results, it is found that the CA-g-CS conjugate film has an excellent effect on the biological activity and fresh-keeping effect of prawns, so it is speculated that CGA may also have great potential in the application of packaging materials (123).

Additionally, some scholars have found that the use of phenolic compounds extracted from sunflower shells to prepare anti-oxidant starch food packaging materials or coatings also has a vast market prospect. It has been discovered that CGA is the primary anti-DPPH free radical active substance in the extracted phenolic compounds. Adding 1–2% sunflower hull extract can produce starch films with high anti-oxidant capacity, and all films exhibit good performance. Oxygen barrier performance and water vapor performance (124). Furthermore, the preparation of CGA-containing CS/polycaprolactone (PCL) electrospun nanofibers (CGA@HNTs/PCL/CS) using electrospinning as a carrier has also had broad applications in the field of food packaging. Market prospects PCL/CS nanofibers, because the addition of CGA@HNTs makes the fibers continuous and uniform, the melting temperature (Tm) and melting enthalpy (ΔHm) also increase, and the thermal stability is improved. Simultaneously, the nanofibers are hydrophilic after adding CGA@HNTs, and the strong hydrophilic surface may reduce bacterial adhesion, and then effectively extend the shelf life of food (125).

### Functional Food Ingredients

In the last few years, natural anti-oxidant compounds have been incorporated into the design of functional foods to prevent many diseases related to oxidative stress, such as cardiovascular and neurodegenerative diseases, diabetes, and cancer. The idea has proved to be very feasible. Sea fennel or sea parsley, is an edible halophilic plant, abundant in the Mediterranean and Atlantic coasts. In ancient times, sailors used this plant to prevent scurvy, and the main compound that the plant is rich in is CGA, so we speculate that CGA can be used as a functional factor in the development of functional foods (126, 127). Additionally, some scholars have established a CGA-glutamic acid (Glu) heating model system (HMS) to evaluate the *In vitro* anti-oxidant and aldose reductase inhibitory effects of new compounds produced after heating at 120°C for 4 h. The mouse lens aldose reductase (RLAR) inhibition rates of the new compounds 1 and 2 derived from heat treatment at 10.0, 1.0, and 0.5-μg/mL were 99.47, 53.67, and 13.80%, respectively. The scavenging rate of DPPH free radicals is better than its precursor CGA. The structure of the compound varies with amino acids. Based on this feature, we speculate that CGA and amino acid HMS can be used as a functional food material for diabetic patients (128).

Barbecuing is widely loved worldwide. It is well-known that grilling requires high temperature treatment, and its range usually exceeds 200°C. However, meat products will promote the formation of carcinogenic heterocyclic amines (HAs) under high temperature processing. Related studies have shown that CGA and HAs key precursors glucose, ribose, fructose, isoleucine, valine, and lysine has a competitive chemical reaction, significantly inhibiting the formation of IQx, 8-MeIQx, Norharman, Harman, and PhIP, The inhibition rates are as high as 13.74–36.64, 49.81–55.85, 59.85–94.18, 73.53–89.92, and 66.32–100%, which restrict the generation of HAs and provide research directions for food safety (129). According to reports, adding mulberry leaves and fruits to honey can enrich its polyphenol compounds and improve its health characteristics. Honey is a controversial ingredient for diabetes, but it has been confirmed that mulberry leaves and mulberry inhibit the activity of α-amylase, and as the content of mulberry leaves increases, the contents of α-GLU and β-galactosidase (β-GAL) in honey also increases. Combining honey with products that can significantly lower blood sugar levels like mulberries may become a modern healthy sweetener for diabetics. HPLC-MS analysis showed that CGA is the main phenolic compound in mulberry leaves (130).

### Prebiotics

Another area of development in food research is prebiotics, which are food ingredients that stimulate the growth of beneficial bacteria in the human gut microbiota (60). Lactic acid bacteria (LAB), including members of the genera *Lactobacillus, Lactococcus, Leuconostoc, Pediococcus*, and *Streptococcus*, are generally recognized as important members of the gastrointestinal (GI) microbiota of humans and animals. *Lactobacillus gasseri*, a LAB, which is used as a starter strain in the production of various fermented dairy products, grows poorly in milk and milk-based media without any supplementation. Therefore, the concept of a plant-based food additive that helps to promote the growth of probiotics was born. Related investigators investigated synbiotic interactions between *Lactobacillus gasseri* strains and *Cudrania tricuspidata* (CT) wood, a potential natural prebiotic source. The results showed that the proteolytic activity in milk was also significantly increased (*P* < 0.05) after 48 h of culture and fermentation when the cell count of the fermented milk supplemented with CT increased from 1.03 to 1.56 log CFU/mL. The contents of neochlorogenic acid, CGA and caffeic acid decreased in the fermented milk detected by CT, and the level of 3,4-dihydroxy cinnamic acid increased, which indicated that endogenous phenolic compounds such as neochlorogenic acid, CGA and caffeic acid Utilized and metabolized to other compounds by *Lactobacillus gasseri* during fermentation. Based on the above conclusions, we can determine that CT can be used as a good source of prebiotics to promote the growth of probiotics, and the main role may be polyphenols such as CGA and caffeic acid (131).

Other researchers also investigated the prebiotic properties of CT and *Morus alba L. leaves* (MA) in the production of yogurt. The results showed that both plants significantly accelerated the acidification of yogurt, and the acidification rate increased by 57 and 75%, respectively. Similarly, hydroxycinnamic acids in plant extracts, such as neochlorogenic acid, CGA, and caffeic acid, are degraded and metabolized during yogurt fermentation (132). In an experiment evaluating the effect of different coffee types, roast levels, and decaffeination on the growth of probiotics *in vitro*, it was also found that coffees with higher levels of CGA, tested *Lactobacillus rhamnosus, Lactobacillus acidophilus*, animal *bile Fidobacteria, Bifidobacterium animalis subsp*. *Lactobacillus* had a higher growth state. This may be due to the fact that *Bifidobacterium* species and *Lactobacillus* species possess an enzyme called cinnamoyl esterase, which can hydrolyze CGA to produce caffeic acid and quinic acid, which in turn promote the growth of both species (133). However, these studies only were *in vitro* test. Studies in humans are needed to evaluate the full potential of chlorogenic acid as a prebiotic.

### Other

Additionally, CGA is used in chicken feed to change the oxidative stress and damage of the intestinal brush border membrane when the ambient temperature is too high (134). Heat stress may accelerate the glycolysis and metabolism of broiler chickens after death, decreasing pH after death and an increase in meat cooking loss. When a certain amount of CGA is added, perhaps the chicken's anti-oxidant capacity increases, and the MDA and carbonyl content reduces. Additionally, the control of the anti-oxidant enzyme system pathway Nrf2 and the expression of downstream enzyme proteins such as SOD, GSH, and CAT have also been significantly increased. At the same time, CGA has also increased the content of polyunsaturated fatty acids (PUFA) in heat-stressed broilers. The main reason may be the increase in the content of C18:2n-6, C18:3n-3, and C20:3n-6. This feature not only enhances the nutritional value of chicken, but also gives the chicken a good taste (135). Additionally, related studies have also shown that adding CGA to pig feed can improve the lipid peroxidation caused by long-term feeding of polyunsaturated fatty acids in vegetable oils, such as oxidized corn oil and the overload of pig viscera. CGA reverses the oxidation of myocardial sarcoplasmic reticulum Ca2+-ATP by oxidized fats, increases the final pH value of pork, and improves the growth performance of pigs and the quality of pig meat after death (136).

In addition, CGA has been widely used in a variety of drugs because of its various biological activities such as antioxidant, anti-inflammatory, antibacterial, antiviral, and hypoglycemic. The “Drug Standards” of the Ministry of Health of China include 170 kinds of Chinese patent medicines with heat-clearing and detoxifying, antibacterial and anti-inflammatory properties, all of which contain CGA as the main ingredient. In the production of Shuang-huang-lian Oral Liquid (137), Re-du-ning Injection (138), Yin-huang Granules (139), and other drugs, CGA has been used as one of the Chinese medicine indicators for quality control. It is worth mentioning that CGA has been identified to be a potential drug for cancer and was approved by the China Food and Drug Administration (CFDA) first for phase I (NCT02728349, Apr. 2016) and then phase II (NCT03758014, Nov. 2018) clinical trials in glioma patients (38, 140).

In late 2019, the outbreak of the novel coronavirus (COVID-19) was declared a Public Health Emergency of International Concern (PHIC) by the World Health Organization. However, the current research and development of virus vaccines lag far behind, and there is an extreme lack of effective therapeutic drugs against the virus in clinical practice. In view of the excellent role of Mongolian medicine in SARS, H1N1, H7N9, and other infectious diseases, as well as the high homology of COVID-19 and SARS virus gene sequences, as well as the pathogenesis characteristics, clinical manifestations and potential therapeutic targets of the two great similarities. Yu et al. used network pharmacology and molecular docking technology to explore the blocking of Mongolian medicine Forsythia and honeysuckle (main components forsythin and CGA) on the binding of S-protein of COVID-19 to angiotensin-converting enzyme 2 (ACE2) in humans effect, the two active substances bind to Gln325/Glu329 and Gln42/Asp38 of ACE2 in the form of hydrogen bonds. The docking energy is small, the binding is more stable, and the binding of the new coronavirus S-protein and ACE2 is effectively blocked at the molecular level. The content of chemical components, the lack of understanding of viruses and diseases, and the limitations of molecular docking itself, the results obtained need further verification (141).

## Discussion

CGA is a phenolic acid compound that exists largely in nature. After continuous theoretical discussion and experimental research by many scholars, we have now clarified the structure of the CGA molecular formula and its role in anti-oxidation, liver and kidney protection, anti-bacterial, anti-tumor, and regulation of glucose metabolism as well as lipid metabolism, anti-inflammatory, and biological activity of protecting the nervous system. Through the gradual exploration of several biological activities, we have shown the effects of CGA on different species, organs, tissues, signal pathways, gene regulation, protease inhibition, growth cycle, and other levels. We also found that CGA has been widely used in the food field and has produced a significant influence. In addition to the medical field, CGA acts as a drug delivery material (142, 143), participates in developing new drugs (144, 145), etc. In animal husbandry, CGA is added to livestock feed to regulate intestinal flora, and increase the preservation period of animal sperm (146, 147), etc. CGA is often involved in cosmetics and daily chemical products due to of its good anti-oxidant and anti-bacterial properties (148, 149). We have summarized the biological activity mechanism of CGA and its application in food, and provided a convenient way for scholars studying this field, in order to make a certain contribution to the subsequent discovery of biological activity and the broadening of the field of CGA.

Simultaneously, we found that there are still some issues to be solved in related fields, such as the mechanism and application of CGA's biological activity:

Experiments have confirmed that CGA plays an essential role in protecting the liver (150), but a recent experiment has shown that in patients with NAFLD and type 2 diabetes, supplementation of 200-mg/d CGA for six consecutive months is effective. Non-invasive markers such as hepatic steatosis, fibrosis, and inflammation did not have a significant effect (151). Therefore, it is necessary for us to explore further the pharmacological dose and safety of CGA with a higher dose and a longer time.

It is mentioned in the application that CGA and amino acids will obtain new compounds after heat treatment. However, the mechanism by which CGA adds amino acids during heating to form active compounds is still unclear. Research on the structure and formation mechanism of active compounds during heating also needs to be explored (128).

Because the molecular structure of CGA contains ester bonds, unsaturated double bonds, and unstable polyphenol structures, it is easy to undergo isomerization through the migration of intramolecular ester groups. CGA extracted from natural plants is often a mixture, which leads to the extraction and purification of CGA with difficulty. In addition, CGA is easily oxidized and decomposed when exposed to heat and light, with poor stability and low solubility. It is necessary to avoid high temperature, strong light, and prolonged heating during the extraction process. It cannot be stored for a long time and can react with almost all components in organisms, which may lead to poor Influence and other factors limiting the large-scale application of CGA in actual production (152).

Studies have revealed that CGA reacts with amino acids and protein side chains under alkaline conditions to produce green trihydroxybenzoacridine (TBA) derivatives. Increasing the pH value from five to nine will cause the color to change from yellow to blue-green. This phenomenon is more likely to occur in baked foods. Consumers' acceptance of food is positively correlated with color. Studies have shown that decreasing the production of TBA can be achieved by using of low pH and low moisture sweeteners, while high greening can be achieved by using of high pH and high-moisture sweeteners (153). But how to ensure that CGA can play an anti-oxidant effect in food, and ensure that it does not affect the visual perception of food is a direction worth exploring at present.

Polyphenols are easily oxidized into oligomers and polymers under alkaline conditions. The materials involved in the preparation are not suitable for long-term storage. Oral absorption also has lower bioavailability, such as first-pass elimination effects. Researchers have proposed microencapsulation (154, 155), yeast and bacteria encapsulation, quaternary ammonium salt cross-linked cationic starch adsorption, and other methods to prevent the rapid disappearance of CGA anti-oxidant properties (156). Additionally, whether we can develop a more efficient and low-cost protection method, so that CGA can be widely used in more fields, it still needs to be further explored.

Applying the polyhydroxyl structure of CGA to protein modification can improve the emulsification system's oxidative stability and emulsion stability. It can also change the structure of allergens and reduce their activity. However, studies have discovered that CGA combined with protein, thermal stability will be reduced (117). Provides a new insight into the production of CGA and the maintenance of its health benefits for dairy beverage companies.

Collectively, numerous studies have demonstrated that CGA is a natural health-beneficial compound, based on its biological effects and potential application in the food field. This review may help advance the development of CGA in functional foods and nutritional formulations, as well as promote it as a newly approved food additive, which of course more experiments are also needed to confirm that CGA has better substitution benefits than traditional additives. We hope that CGA will be used in future development and application, not only in the food field but also in a variety of technical fields including pharmaceuticals, materials engineering, fine chemistry, the manufacture of semiconductors, textiles, and special machines (157).

## Conclusion

CGA plays an active role in the protection of human health, food taste, and food safety, or life-related needs. Our biological activities of CGA, include anti-oxidant, liver and kidney protection, anti-bacterial, anti-tumor, and sugar regulation. Metabolism and lipid metabolism, anti-inflammatory, and protection of the nervous system were discussed. Simultaneously, we also discovered that CGA has been widely used in the food field and has a massive impact. For example, CGA can be used as a food additive, extend food storage time, and change food composition characteristics, be a good auxiliary for food packaging materials and functional food raw materials, etc. However, in actual research and application, CGA still has many issues that need to be solved urgently, including stability, potential toxicity, bioavailability, and expansion of application fields, etc., which require further excavation and exploration. Therefore, we believe that we still have to go deeper in CGA research to make it usable in our daily lives.

## Author Contributions

YChe, SL, and CP designed the study. LW drafted the manuscript. XP, LJ, YChu, SG, XJ, and YZ were major contributors in reviewing the manuscript. Based on the contributions, LW was listed as the first author while YChe, SL, and CP were the correspondences. All authors read and approved the final manuscript.

## Funding

This study was funded by the National Natural Science Foundation of China (NSFC) by Projects (Nos. 81891012 and U19A2010) and Natural Science Foundation of Sichuan Province (No. 2022NSFSC0577).

## Conflict of Interest

The authors declare that the research was conducted in the absence of any commercial or financial relationships that could be construed as a potential conflict of interest.

## Publisher's Note

All claims expressed in this article are solely those of the authors and do not necessarily represent those of their affiliated organizations, or those of the publisher, the editors and the reviewers. Any product that may be evaluated in this article, or claim that may be made by its manufacturer, is not guaranteed or endorsed by the publisher.
